# Pan-cancer diagnostic consensus through searching archival histopathology images using artificial intelligence

**DOI:** 10.1038/s41746-020-0238-2

**Published:** 2020-03-10

**Authors:** Shivam Kalra, H. R. Tizhoosh, Sultaan Shah, Charles Choi, Savvas Damaskinos, Amir Safarpoor, Sobhan Shafiei, Morteza Babaie, Phedias Diamandis, Clinton J. V. Campbell, Liron Pantanowitz

**Affiliations:** 1 Huron Digital Pathology, St. Jacobs, ON Canada; 2 Kimia Lab, University of Waterloo, Waterloo, ON Canada; 3 Vector Institute, MaRS Centre, Toronto, ON Canada; 4 General Hospital/Research Institute (UHN), Toronto, Canada; 5 Stem Cell and Cancer Research Institute, McMaster University, Hamilton, Canada; 6 Department of Pathology and Molecular Medicine, McMaster University, Hamilton, Canada; 7 Department of Pathology, University of Pittsburgh Medical Center, Pittsburgh, PA USA

**Keywords:** Cancer imaging, Data mining, Machine learning

## Abstract

The emergence of digital pathology has opened new horizons for histopathology. Artificial intelligence (AI) algorithms are able to operate on digitized slides to assist pathologists with different tasks. Whereas AI-involving classification and segmentation methods have obvious benefits for image analysis, image search represents a fundamental shift in computational pathology. Matching the pathology of new patients with already diagnosed and curated cases offers pathologists a new approach to improve diagnostic accuracy through visual inspection of similar cases and computational majority vote for consensus building. In this study, we report the results from searching the largest public repository (The Cancer Genome Atlas, TCGA) of whole-slide images from almost 11,000 patients. We successfully indexed and searched almost 30,000 high-resolution digitized slides constituting 16 terabytes of data comprised of 20 million 1000 × 1000 pixels image patches. The TCGA image database covers 25 anatomic sites and contains 32 cancer subtypes. High-performance storage and GPU power were employed for experimentation. The results were assessed with conservative “majority voting” to build consensus for subtype diagnosis through vertical search and demonstrated high accuracy values for both frozen section slides (e.g., bladder urothelial carcinoma 93%, kidney renal clear cell carcinoma 97%, and ovarian serous cystadenocarcinoma 99%) and permanent histopathology slides (e.g., prostate adenocarcinoma 98%, skin cutaneous melanoma 99%, and thymoma 100%). The key finding of this validation study was that computational consensus appears to be possible for rendering diagnoses if a sufficiently large number of searchable cases are available for each cancer subtype.

## Introduction

Digital pathology is the virtual version of conventional microscopy utilized for the examination of glass pathology slides. In recent years, there has been accelerated adoption of digital pathology, whereby pathology laboratories around the world are slowly beginning to trade in their light microscopes for digital scanners, computers, and monitors. As a result, the pathology community has begun to scan many slides resulting in the creation of large databases of whole-slide images (WSIs). The emergence of deep learning and other artificial intelligence (AI) methods and their impressive pattern-recognition capabilities when applied to these digital databases has immensely added to the value proposition of digital pathology^1–3^. Computerized operations, such as segmentation of tissue fragments and cell nuclei, and classification of diseases and their grades become possible after pathology slides are digitized. These operations could assist with many diagnostic and research tasks with expert-like accuracy when trained with the proper level of labeled data^4^. The majority of recent studies in digital pathology have reported the success of supervised AI algorithms for classification and segmentation^4–7^. This overrepresentation compared with other AI algorithms is related to the ease of design and in-lab validation to generate highly accurate results. However, compared with other methods of computer-vision algorithms, AI-based image search and retrieval offers a new approach to computational pathology.

Content-based image search^8–11^ implies that the input for search software is not text (e.g., disease description in a pathology report), but rather the input is an image such that the search and retrieval can be performed based on image pixels (visual content). Content-based image search is inherently unsupervised, which means that its design and implementation may not need manual delineation of a region of interest in the images^12–14^. More importantly, image search does not make any direct diagnostic decision on behalf of the pathologist; instead, it searches for similar images and retrieves them along with the corresponding metadata (i.e., pathology reports), and displays them to the pathologist as decision support.

Variability in the visual inspection of medical images is a well-known problem^15–17^. Both inter- and intra-observer variability may affect image assessment and subsequently the ensuing diagnosis^18–21^. A large body of work have reported high rates of diagnostic inaccuracy as a result of major discordance among participating physicians with respect to case target diagnoses, and propose a combination of “routine second opinions” and “directed retrospective peer review”^22–24^. As most proposed AI-driven solutions for digital pathology mainly focus on the concept of classification, it appears that algorithmic decision-making may not necessarily contribute to supporting concordance by providing a framework for consensus building. Most capable classification schemes trained with immense effort are supposed to be used for triaging cases in the pathology laboratory, and not for direct assistance in the pathologist’s office^4^. In contrast, instantly retrieving multiple diagnosed cases with histopathologic similarity to the patient’s biopsy about to be diagnosed offers a new generation of decision support that may even enable “virtual” peer review.

Content-based image retrieval (CBIR) systems have been under investigation for more than two decades^25–27^. Recently, deep learning has gained a lot of attention for image search^28–30^. While CBIR systems of medical images have been well researched^11,31–33^, only with the emergence of digital pathology^34,35^ and deep learning^3,36,37^ has research begun to focus on image search and analysis in histopathology^2,38–40^. In the past 3 years, an image search engine called *Yottixel* has been designed and developed for application in pathology^32,41–43^. Yottixel is a portmanteau for *one yotta pixel* alluding to the big-data nature of pathology images. The underlying technology behind Yottixel consists of a series of AI algorithms, including clustering techniques, deep networks, and gradient barcoding. By generating a “bunch of barcodes” (BoB) for each WSI, digitized pathology slides can be indexed for real-time search. In other words, the tissue patterns of a WSI are converted into barcodes, a process that is both storage-friendly and computationally efficient. In this paper, we report the outcome of a comprehensive validation of the Yottixel search engine. We used WSI data from The Cancer Genome Atlas (TCGA) repository provided by the National Cancer Institute (NCI)/National Institutes of Health (NIH). Almost 30,000 WSI files of 25 primary anatomic sites and 32 cancer subtypes were processed by dismantling these large slides into almost 20,000,000 image patches (also called tiles) that were then individually indexed employing ~3,000,000 barcodes. We employ the largest publicly available archive of WSIs to verify the performance of an image search engine for digital pathology.

## Results

### Performance measurement of search engine

In two major series of experiments, we calculated the “accuracy” of image search through “leave-one-patient-out” samplings. Whereas the literature of computer vision focuses on top-n accuracy (if any one of the n search results is correct, then the search is considered be to be successful), we calculated the majority-n accuracy (only if the majority among n search results were correct, the search was considered correct). Specifically, “correct” means that the tumor type (horizontal search) or tumor subtype within a specific diagnostic category (vertical search) was recognized correctly and matched by the majority of identified and retrieved cases. In order to avoid falsification of results through anatomic duplicates, we excluded all WSIs of the patient when one of the WSIs was the query.

#### Horizontal search: cancer-type recognition

The first series of experiments undertaken for all anatomic sites was *horizontal search*. The query WSI is compared against all other cases in the repository, regardless of anatomic site categorization. Of course, the primary anatomic site is generally known, and, in many cases, the cancer type may also be known to the pathologist. Thus, the purpose of the horizontal search (which is for either organ or cancer-type recognition) is principally a fundamental algorithmic validation that may also have applications like searching for origin of malignancy in case of metastatic cancer.

**Fig. 1:**
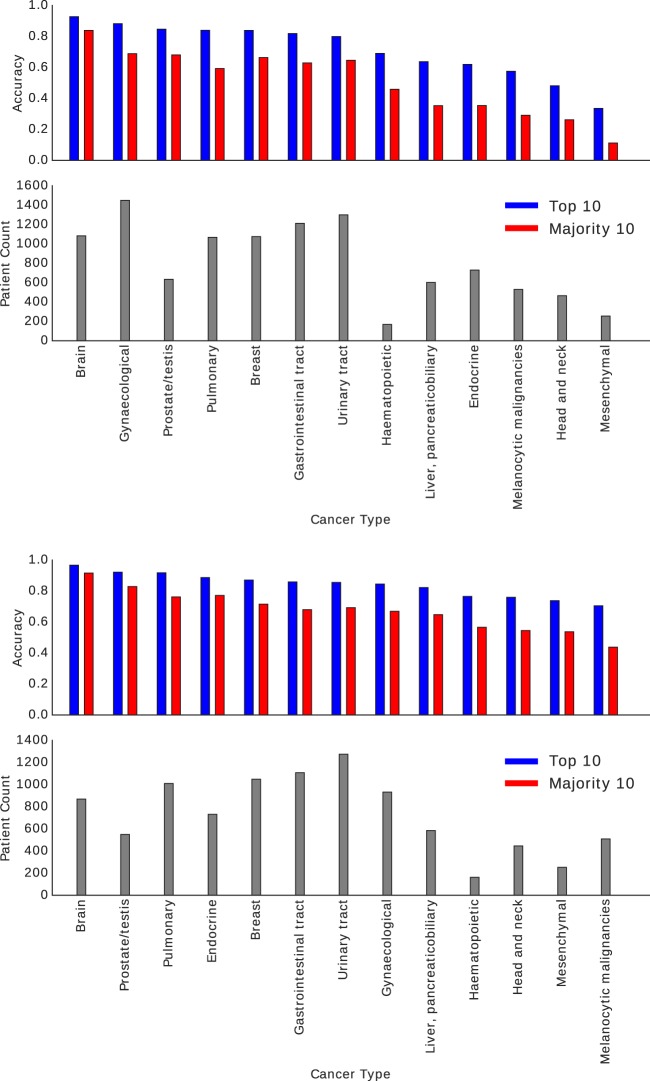
Horizontal search for frozen sections (top) and permanent diagnostic slides (bottom). Details are demonstrated in Tables 1 and 2 in the Appendix.

The results of the horizontal search are depicted in Fig. 1 (see Appendix for details with Table 1 showing results for frozen section and Table 2 for permanent diagnostic slides). All experiments were conducted via “*leave-one-patient-out*” validation.

**Table 1:** Results for cancer-type recognition (horizontal search) among frozen slides.

Tumor type	WSI count	Patient count	Hit rate (%)			Majority-5		Majority-10	
			Top-10	Top-5	Top-3	Accuracy	Recall	Accuracy	Recall
Brain	1797	1083	97.44	95.21	92.76	82.24	86.37	83.86	86.42
Gynecological	2216	1450	97.60	93.50	88.22	67.96	77.03	68.86	78.97
Pulmonary	1634	1068	95.34	90.75	83.90	58.01	65.61	59.30	67.99
Gastrointestinal tract	1947	1212	95.12	87.98	81.86	61.32	68.16	62.86	68.98
Breast	1495	1075	93.44	88.56	83.87	65.61	74.45	66.35	77.46
Prostate/testis	755	634	91.92	87.28	84.63	66.22	74.30	68.07	73.77
Urinary tract	1980	1300	90.25	83.48	79.89	62.67	68.89	64.59	67.83
Endocrine	769	729	84.78	71.39	61.89	30.68	44.08	35.37	43.56
Melanocytic malignancies	532	529	83.83	68.79	57.51	25.93	39.85	29.13	39.85
Liver, pancreaticobiliary	659	602	81.48	73.29	63.73	30.34	44.61	35.35	43.55
Hematopoietic	181	169	78.45	73.48	69.06	44.19	55.25	45.85	49.17
Head and neck	663	465	70.88	57.16	48.11	22.32	29.56	26.24	27.75
Mesenchymal	259	255	56.37	42.85	33.59	06.17	16.22	11.19	15.44

Every whole-slide image was compared with all other slides in the repository regardless of the primary site. The table is sorted based on Top-10 hit rates. The accuracy and recall (sensitivity) for majority-5 and majority-10 among search results are provided as well.

**Table 2:** Results for cancer-type recognition (horizontal search) among diagnostic slides.

Tumor Type	WSI count	Patient count	Hit rate (%)			Majority-5		Majority-10	
			Top-10	Top-5	Top-3	Accuracy	Recall	Accuracy	blackRecall
Brain	1692	870	98.99	97.81	96.69	91.37	94.33	91.60	94.80
Pulmonary	1109	1011	98.46	96.12	91.70	75.83	84.58	76.19	86.29
Prostate/testis	701	550	97.43	94.86	92.15	80.31	86.73	82.88	85.31
Breast	1116	1049	95.96	91.57	87.09	70.87	78.79	71.50	78.61
Gastrointestinal tract	1144	1108	95.54	90.73	85.83	65.12	74.25	67.91	74.59
Urinary tract	1374	1275	95.41	90.82	85.51	66.01	74.56	69.21	73.84
Gynecological	1039	933	95.28	90.37	84.50	63.71	73.40	66.89	74.88
Endocrine	936	732	94.55	91.88	88.67	73.93	81.45	77.13	81.34
Liver, pancreaticobiliary	618	585	93.85	87.37	82.20	63.75	70.32	64.72	70.81
Head and neck	466	446	90.55	82.40	75.96	49.14	60.94	54.50	57.94
Melanocytic malignancies	551	509	88.20	79.31	70.41	37.20	51.91	43.73	52.09
Mesenchymal	594	253	87.37	80.63	73.73	50.84	61.78	53.70	64.14
Hematopoietic	221	163	84.61	81.44	76.47	52.03	64.25	56.56	61.09

Every whole-slide image was compared with all other slides in the repository regardless of the primary site. The table is sorted based on Top-10 hit rates. The accuracy and recall (sensitivity) for majority-5 and majority-10 among search results are provided as well.

The following observations can be made from the results:

- Provided there are *sufficient* number of patients, we observed that the more we retrieve the more likely it was to achieve the right diagnosis: top-10 is better than top-5, and top-5 is better than top-3.
- General top-n accuracy that is common in the computer-vision literature (top-3, top-5 and top-10 column in Tables 1 and 2) show high values, but may not be suitable in the medical domain as it considers the search to be a success if at least one of the search results has the same cancer type as the query image.
- The majority vote among top-n search results appears to be much more conservative and perhaps more appropriate, as it only considers a search task as successful if the majority of top-n search results show the same cancer type as the query image (majority-5 and majority-10 columns in Tables 1 and 2).
- With some exceptions, a general trend is observable that the more images/patients are available the higher the search-based consensus accuracy. The number of cases positively correlated with the majority-vote accuracy for both frozen sections and permanent diagnostic slides.

#### Vertical search: correctly subtyping cancer

In the second series of experiments, we performed *vertical search*. Given the primary site of the query slide we confined the search only to WSIs from that organ. Hence, the goal of the vertical search was to recognize the cancer subtype. For this purpose, only those primary anatomic sites in the data set with at least two possible subtypes were selected. Sample retrievals are illustrated in Appendix Fig. 2. The results for “leave-one-patient-out” validation are depicted in Figs 3 and 4 (details in Appendix, Table 3 for frozen sections and Table 4 for diagnostic slides).

**Fig. 2:**
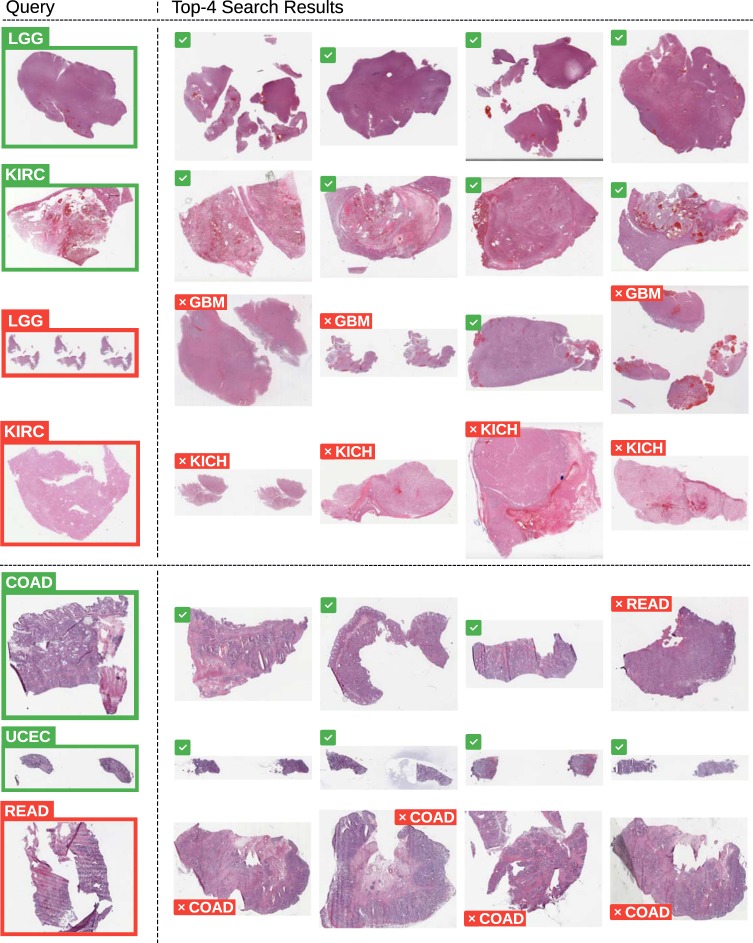
Sample retrievals for cancer subtype categorization through majority votes. The top four slides are of permanent diagnostic slides whereas the bottom three slides are of frozen section slides. The misclassified and successful queries are marked with red and green boundaries, respectively (for abbreviations, see Table 5).

**Fig. 3:**
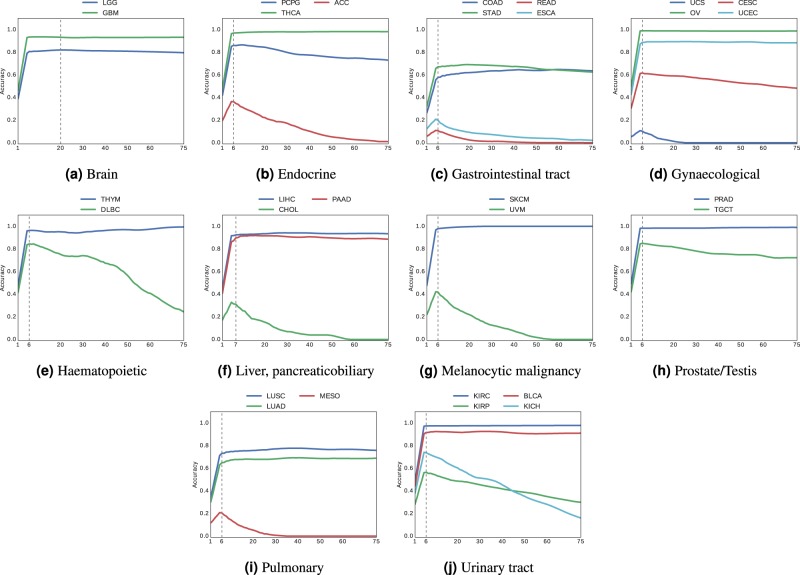
Accuracy of vertical search for frozen sections. Vertical search in frozen sections slides from different anatomic sites (**a**–**j**) with at least two cancer subtypes.

**Fig. 4:**
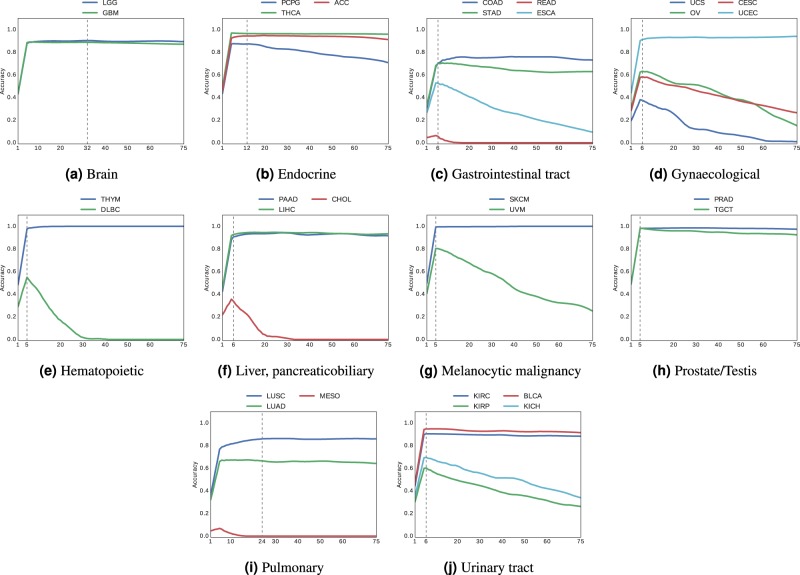
Accuracy of vertical search for diagnostic slides. Vertical search in permanent diagnostic slides from different anatomic sites (**a**–**j**) with at least two cancer subtypes.

**Table 3:** Accuracy and recall (sensitivity) for cancer subtype identification (vertical search) among frozen section slides.

Tumor type	WSI count	Patient count	Majority-5		Majority-10		Majority-20	
			Accuracy	Recall	Accuracy	Recall	Accuracy	Recall
*Brain*								
GBM	1102	582	94.19	94.19	92.74	94.37	92.92	93.65
LGG	695	501	82.58	82.59	80.28	83.02	81.00	83.31
*Endocrine*								
ACC	81	81	45.67	46.91	28.39	48.15	20.98	35.80
PCPG	174	170	85.63	86.78	86.20	89.66	83.90	86.78
THCA	514	478	97.08	97.67	97.47	98.44	97.85	98.83
*Gastrointestinal tract*								
COAD	830	449	63.73	69.40	56.62	74.10	60.00	78.43
ESCA	166	165	25.90	31.33	12.04	23.49	09.03	15.66
STAD	623	428	71.10	74.48	65.48	80.42	67.41	81.70
READ	328	170	14.32	19.21	05.48	14.63	02.13	8.54
*Gynecological*								
OV	1184	586	99.07	99.24	98.98	99.16	98.81	99.16
CESC	298	291	64.42	68.12	59.06	65.44	58.05	63.42
UCS	49	49	10.20	12.24	04.08	12.24	02.04	2.04
UCEC	685	524	90.07	90.80	89.05	90.80	89.34	91.68
*Hematopoietic*								
DLBC	57	45	91.22	91.23	80.70	87.72	73.68	78.95
THYM	124	124	97.58	97.58	95.16	97.58	95.16	95.97
*Liver, pancreaticobiliary*								
LIHC	392	370	93.36	93.88	92.60	94.64	93.62	94.90
CHOL	51	51	35.29	45.10	19.60	47.06	13.72	27.45
PAAD	216	181	91.66	91.67	90.74	93.52	90.74	93.98
*Melanocytic malignancies*								
SKCM	463	460	98.70	98.49	98.48	98.92	99.56	99.78
UVM	69	69	46.37	46.38	31.88	39.13	18.84	27.54
*Prostate/testis*								
TGCT	155	149	86.45	87.74	83.87	85.81	81.29	85.81
PRAD	600	485	98.33	98.33	98.33	98.33	98.50	98.67
*Pulmonary*								
LUSC	745	485	78.25	78.79	70.87	77.99	73.42	77.72
LUAD	806	500	68.23	69.11	64.14	71.34	66.12	70.84
MESO	83	83	27.71	32.53	14.45	26.51	03.61	21.69
*Urinary tract*								
BLCA	420	401	92.85	94.29	90.95	94.29	90.95	95.00
KICH	138	88	78.26	81.16	68.11	77.54	57.24	73.19
KIRC	1055	529	97.81	97.91	97.25	98.20	97.63	98.20
KIRP	367	282	62.12	67.30	51.22	63.76	47.13	58.86

Only those primary sites were considered for vertical search which had at least two subtypes in the repository. A positive correlation of 0.57 was measured between the number of patients and the highest accuracy.

**Table 4:** Accuracy and recall (sensitivity) for cancer subtype identification (vertical search) among permanent diagnostic slides.

Tumor type	WSI count	Patient count	Majority-5		Majority-10		Majority-20	
			Accuracy	Recall	Accuracy	Recall	Accuracy	Recall
*Brain*								
GBM	851	381	91.18	91.30	87.89	90.01	88.13	89.42
LGG	841	489	89.77	89.54	88.58	90.61	89.17	91.20
*Endocrine*								
ACC	227	56	93.83	93.39	94.27	94.71	94.71	96.92
PCPG	196	176	88.77	88.78	85.71	90.31	84.18	89.29
THCA	513	500	97.66	97.86	96.68	96.89	96.49	96.70
*Gastrointestinal tract*								
COAD	436	428	76.14	82.00	69.72	86.00	74.31	90.00
ESCA	157	155	59.87	69.43	45.22	64.33	39.49	55.41
READ	157	156	10.19	12.20	00.63	3.66	00.00	0.61
STAD	394	369	75.12	79.19	67.76	81.73	67.00	83.25
*Gynecological*								
UCEC	566	505	92.22	93.95	91.69	95.29	92.75	95.97
CESC	277	267	62.45	64.08	54.51	64.79	49.09	58.80
UCS	90	56	42.22	51.11	32.22	48.89	27.77	40.00
OV	106	105	66.98	67.92	59.43	67.92	51.88	62.26
*Hematopoietic*								
DLBC	43	43	58.13	53.49	37.20	58.14	16.27	27.91
THYM	178	120	98.87	98.88	99.43	99.44	100.00	100.00
*Liver, pancreaticobiliary*								
CHOL	39	39	43.58	43.59	25.64	35.90	02.56	17.95
LIHC	378	364	93.65	94.21	93.65	94.74	94.44	95.00
PAAD	201	182	91.04	93.53	92.03	95.02	93.03	99.00
*Melanocytic malignancies*								
UVM	80	80	83.75	83.75	77.50	82.50	68.75	72.50
SKCM	471	429	99.57	99.58	99.57	99.79	99.57	99.79
*Prostate/testis*								
TGCT	254	149	99.21	99.61	96.85	98.82	96.06	96.06
PRAD	447	401	98.43	98.21	98.21	98.66	98.43	98.43
*Pulmonary*								
LUAD	520	465	70.96	71.35	63.26	72.31	64.42	72.50
MESO	86	74	08.13	12.79	02.32	8.14	00.00	1.16
LUSC	503	472	81.70	82.31	78.13	84.10	83.30	88.47
*Urinary tract*								
BLCA	454	384	95.81	96.93	94.27	95.83	93.61	95.83
KIRC	516	511	91.66	93.02	90.11	92.44	89.53	92.64
KICH	108	108	75.92	82.41	66.66	74.07	59.25	70.37
KIRP	296	272	67.22	72.64	53.04	67.91	48.31	64.86

Only those primary sites were considered for vertical search which had at least two subtypes in the repository. A positive correlation of 0.49 was measured between the number of patients and the highest accuracy.

Looking at the results of Figs. 3 and 4 (Tables 3 and 4), we can observe the following:

- For both frozen sections and permanent diagnostic slides, we continue to see a general trend whereby “*the more patients the better*” with both positive exceptions (KICH with 196 patients, and PCPG with 179 patients in Table 3) and negative exceptions (LUAD with 520 patients in Table 4).
- With majority-vote accuracy values for frozen sections (Table 3) in excess of 90% (KIRC, GBM, COAD, UCEC, PCPG), a search-based computational consensus appear to be possible when a large number of evidently diagnosed patients are available.
- With majority-vote accuracy values for diagnostic slides (Table 4) in excess of 90% (GBM, LGG, UCEC, KIRC, COAD, ACC, PCPG), a search-based computational consensus appear to be possible when a large number of evidently diagnosed patients are available.
- In most cases, it appeared that taking the majority of the top-7 search results provided the highest accuracy in most cases. However, the accuracy dropped drastically for subtypes with a small number of patients as we retrieved more and more images beyond six slides, as the majority in such cases were taken from incorrect cases (we do not filter any result; no threshold is used; hence, all search results are considered as valid results).
- Based on all observations, it seems that there is a direct relationship between the number of diagnosed WSIs in the data set and achievable consensus accuracy. For vertical search, we calculated positive correlations of $0.5456$ for frozen sections (Table 3) and $0.5974$ for permanent diagnostic slides (Table 4). This trend was more pronounced for horizontal search with positive correlation of $0.7780$ for frozen sections slides (Table 1), and $0.7201$ for permanent diagnostic slides (Table 2).
- In addition, the Cox-Stuart trend test^44^ was used to check the upward monotonic trend of accuracy with respect to patients number. Having an increasing trend is considered as the null hypothesis for this test. The $p$-values for the horizontal (vertical) search are 1 (0.9991) and 0.9844 (0.9713) for frozen and diagnostic slides, respectively. Since the $p$-values are greater than the significance level (0.05), the null hypothesis is accepted. Consequently, there is a strong evidence of an upward monotonic trend.

#### Visualization of search results

**Fig. 5:**
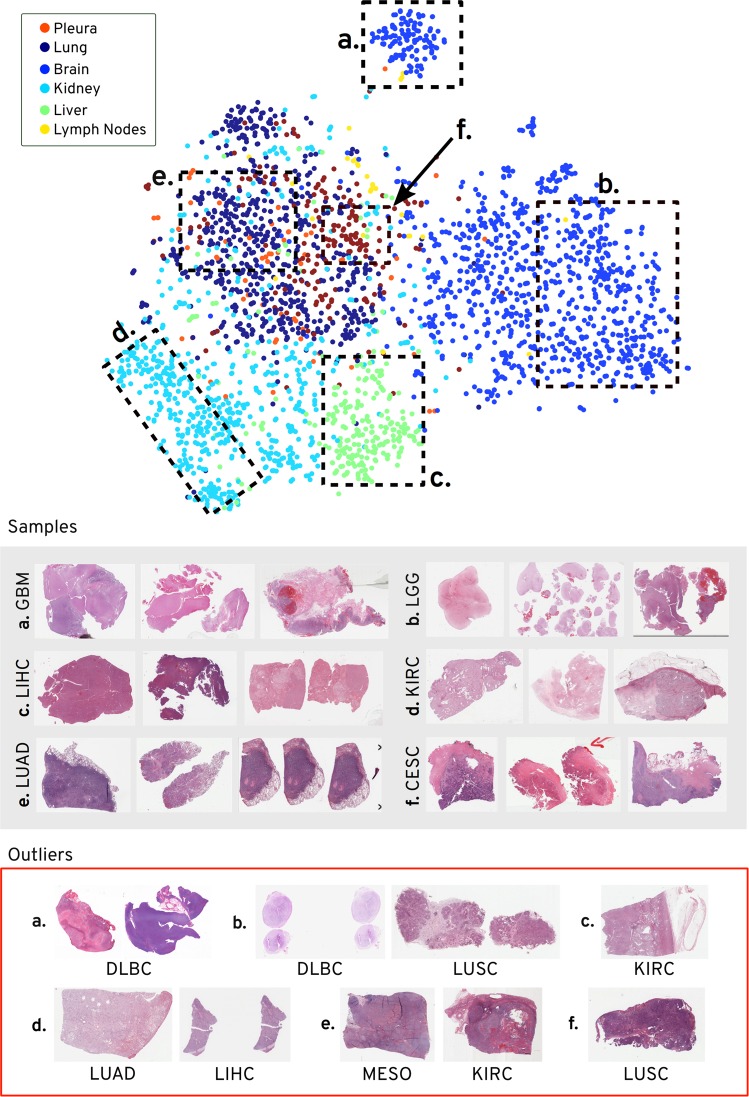
T-distributed Stochastic Neighbor Embedding (t-SNE) visualization of pairwise distances of 3000 randomly selected diagnostic slides from six different primary sites. These primary sites are selected to contain top, average, worst accuracy from the Table 2—lung, brain (top-2), kidney, liver (middle-2), lymph nodes, and pleura (bottom-2). Six different areas containing majority of the points from the same cancer subtype are assigned with unique alphabets—a, b, c, d, e, f. The random slides from the majority cancer subtype within each of the assigned areas are shown in *Samples* box (gray background). The outliers (not belonging to majority the cancer subtype or the primary site) are shown in the outliers box (red outline). For example, area a contains majority of scans from brain with glioblastoma multiforme (GBM), whereas its outliers are from lymph nodes with diffuse large B-cell lymphoma (DLBC). Without any explicit training, our technique maintains the semantic categories within the diagnostic slides as shows by the t-SNE plot of the pairwise distances. The kidney, liver, and brain form different isolated groups whereas lung, pleura, and lymph nodes are intermixed with each other.

Examining best, average, and worst cases for diagnostic slides, we randomly selected 3000 slides and visualized them using the T-distributed Stochastic Neighbor Embedding (t-SNE) method^45^ (see Fig. 5). From this visualization, we can observe that several subtype groups have been correctly extracted through search (see groups *a* to *f*). We can also observe the presence of outliers (e.g., DLBC in groups *a* and *b*). The outliers may be a product of the resolution of these scans, at least in part. At 20× magnification, for example, recognizing a diffuse large B-cell lymphoma (DLBC) from other large cell, undifferentiated non-hematopoietic tumors may not always be immediately possible for pathologists. This typically requires serial sections examined at multiple magnifications with ancillary studies such as immunohistochemistry.

### The challenge of validating histologic similarity

One of the major benefits of using classification methods is that they can easily be validated; every image belongs to a class or not, a binary concept that can be conveniently quantified by counting the number of correctly/incorrectly categorized cases. It should be noted that through treating the image search as a classifier, we have not only used the primary diagnosis for “objective” evaluation of search results but also we are most likely ignoring some performance aspects of image search as search is a technology inherently suitable for looking at border cases and fuzziness of histologic similarity. The concept of similarity in image search is intrinsically a gradual concept (i.e., cannot be answered with a simple yes/no in many cases) and mostly a matter of degree (very similar, quite dissimilar, etc.). In addition, the similarity (or dissimilarity) between images is generally calculated using a distance metric/measure (in our case the Hamming distance^46^). The histologic similarity as perceived by pathologists may not correspond to tests where we used distance as a classification criterion. In other words, the classification-based tests that we run may be too harsh for search results and ignorant toward anatomic similarities among different organs.

**Fig. 6:**
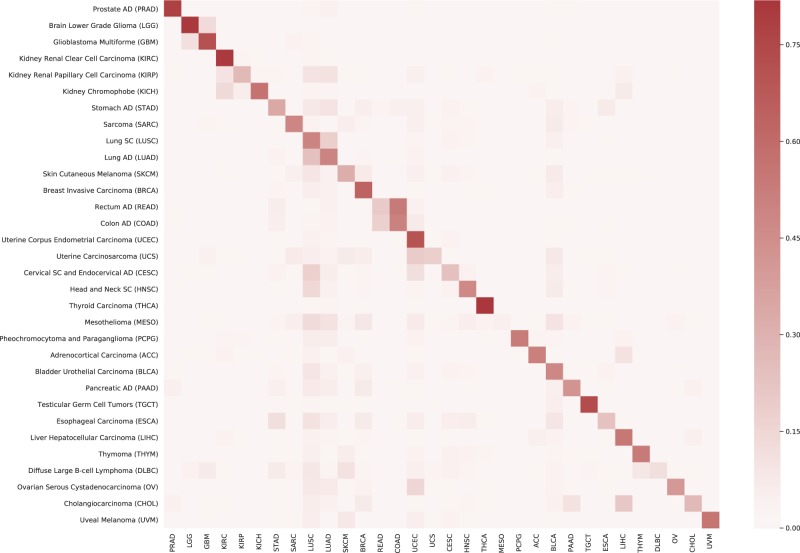
Heatmap of re-scaled relative frequency of matched (red) and mismatched (pale) search results for each diagnosis from permanent diagnostic slides. Re-scaling of frequencies was done through dividing each frequency by the total number of slides for each subtype.

One of the possible ways of examining the performance of the search is to look at the *heatmap*^47^ of the confusion matrix. The values to construct the heatmap can be derived from the relative frequency of every subtype among the top ten search results for a given subtype. A perfect heatmap would exhibit a pronounced diagonal with other cells being insignificant. Figure 6 shows the generated heatmap for all diagnostic subtypes in the data set. The ordering of subtypes along the $y$-axis was done manually. It should be noted that our matching heatmap is not symmetrical like a correlation-based heatmap.

#### Analysis of the heatmap

The pronounced diagonal in Fig. 6 shows that most disease subtypes have been correctly classified as they were very frequently retrieved among the top ten horizontal search results. Other obvious observations:

- MESO is a difficult diagnosis with almost absent diagonal values.
- READ and COAD build a confusion region of four squares; they are confused with each other frequently.
- The same observation can be made for LUAD and LUSC. The vertical values for LUAD and LUSC also show that they are present in many other searches, for instance, when we search for UESC, HNSC, and ESCA.
- LIHC is frequently among the search results for CHOL.
- For PRAD and BRCA we predominantly found PRAD and BRCA images, respectively.

**Fig. 7:**
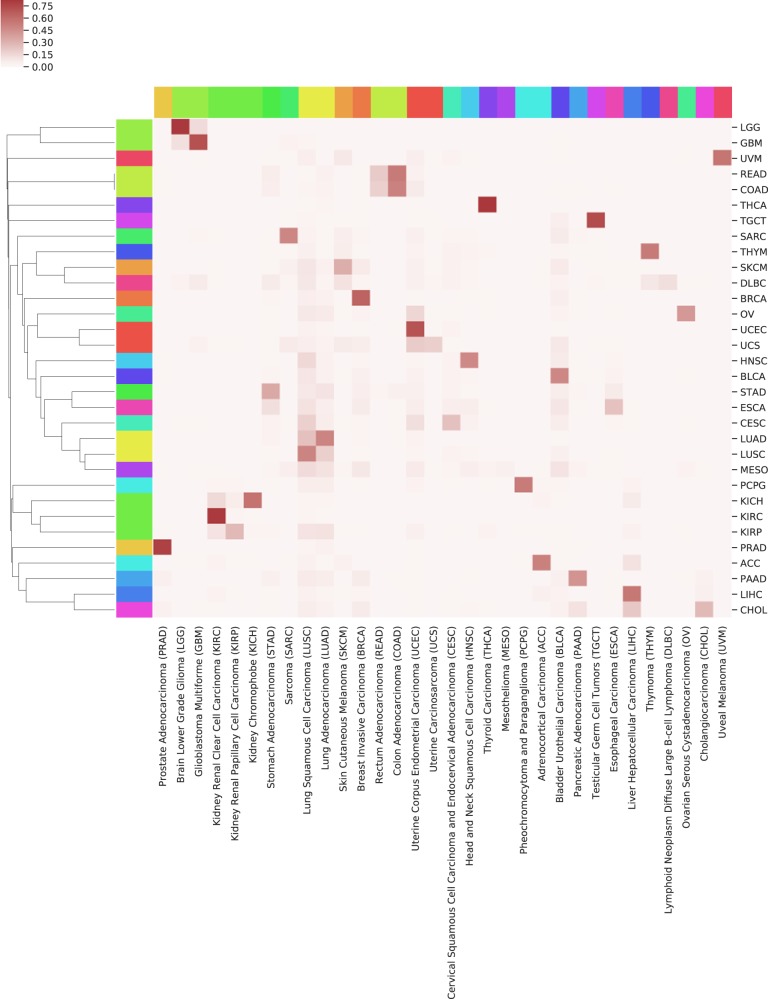
Recognizing structures through clustering. Dendrograms of clustered relative search frequencies.

Of note, the observational analysis of the heatmap alone may be limited. If we cluster (group) the search result frequencies and construct the dendrograms for the relationships in order to create an advanced heatmap, we might more easily discover the benefits of the search (see Fig. 7). From there, we can observe:

- LGG and GBM are both glial tumors of the central nervous system.
- Rectum and colon cancer are gland forming tumors of the colon.
- Both uterine and ovarian carcinoma are grouped under gynecological.
- Gallbladder, stomach, and esophagus are upper gastrointestinal tumors.
- Adenocarcinoma and squamous cell carcinoma are both subtypes of lung tumors.
- Three kidney tumors appear close together.

The errors (i.e., misclassifications) identified were still within the general grouping that the tumor originated from. Hence, from an image search perspective, it suggests that is it good at being close to the site of origin when it makes “classification” errors.

### Chord diagram of image search

**Fig. 8:**
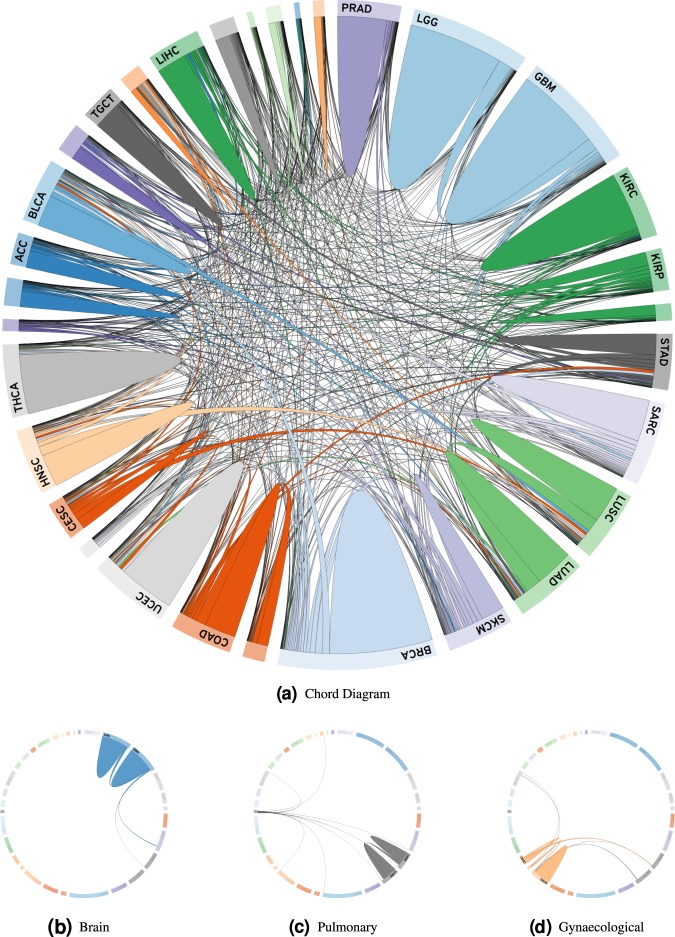
Horizontal search. **a** Chord diagram of horizontal image search for diagnostic slides of the TCGA data set. Sample relations for (**b**) brain (LGG and GBM), (**c**) pulmonary (LAUD, LUSC, and MESO), and (**d**) gynecological (UCEC, UCS, and CESC). The chord diagram can be interactively viewed online: https://bit.ly/2k6g3k1.

We used a chord diagram to further explore retrieved results. A chord diagram is the graphic display of the inter-relationships between numbers in a matrix. The numbers are arranged radially around a circle with the relationships between the data points generally visualized as arcs connecting the numbers/labels^48^. In Fig. 8a, the chord diagram of horizontal search (cancer-type recognition) for 11,579 permanent diagnostic slides of the TCGA data set is illustrated. We can observe the following:

- Adenocarcinomas from several disparate organ systems match (e.g., colon, lung, stomach, and breast). This is not surprising, as adenocarcinomas formed by glandular structures of equivalent grade in most organs are morphologically similar.
- Certain tumors derived from the same organ are related (e.g., LGG and GBM, UCEC and CESC, and kidney RCC and KIRP).
- High-grade tumors from different anatomic locations appear to match (e.g., GBM and sarcoma). This may be attributed to the fact that such high-grade tumors likely display similar morphologic findings (e.g., necrosis).
- Squamous tumors from the head and neck and lung resemble urothelial carcinoma from the urinary bladder. In clinical practice, this differential diagnosis can be morphologically challenging to diagnose, and thus warrants the use of ancillary studies such as immunohistochemistry to determine tumor origin.
- Hepatocellular carcinoma and thyroid carcinoma appear to exhibit the greatest number of matches (eight to nine) to other tumor subtypes. The significance of this finding is unclear.
- The broad relationship demonstrated among certain tumor subtypes is unexpected (e.g., cutaneous melanoma to sarcoma, LUSC, and adenocarcinoma from several organs). Indeed, melanoma is known as the great mimicker in pathology given that these melanocytic tumors can take on many morphological appearances.

One has to emphasize that some relationships depicted in the chord diagram may disappear if distances are normalized and threshold applied. We did not filter any search results. No threshold was used. Hence, all search results were considered. The interactive version of TSNE plot is available online at http://dev1-kimia.uwaterloo.ca:5001/.

## Discussion

The accelerated adoption of digital pathology is coinciding with and probably partly attributed to recent progress in AI applications in the field of pathology. This disruption in the field of pathology offers a historic chance to find novel solutions for major challenges in diagnostic histopathology and adjacent fields, including biodiscovery. In this study, we indexed and searched the largest publicly available data set of histopathology WSIs provided by the NIH/NCI. The question was whether one can build a computational consensus to potentially remedy the high intra- and inter-observer variability seen with diagnosing certain pathology tumors through search in a large archive of previously (and evidently) diagnosed cases. We performed a horizontal search to verify basic recognition capabilities of the image search engine. Furthermore, we performed leave-one-patient-out vertical searches to examine the accuracy of top $n$ search results for establishing a diagnostic majority for cancer subtypes.

The results of this validation study show that building a computational consensus to assist pathologists with “virtual peer review” is possible if large and representative archives of well-characterized and evidently diagnosed cases are available. The ideal size of the data set appears to be in excess of several thousand patients for each primary diagnosis, and is most likely directly related to the anatomic complexity and intrinsic polymorphism of individual tissue types.

Whereas one may need substantial computational power (i.e., a set of high-performance GPUs) to index a large existing repository from scratch, the usage of bunch-of-barcodes idea makes the continuous indexing and search quite feasible for any laboratory, clinic, and hospital.

Since we used a mosaic (a set of patches) to represent and to retrieve WSIs, the search was guided to look for features present in multiple patches to classify the entire WSI. For detailed search, such as mitotic rates and grading applications, one needs a different data set and should also apply single-patch search to look for details. As well, regardless of implementation (e.g., onsite versus cloud), the validated search technology is completely safe toward patient-sensitive information as the barcodes do not contain any reversible information that could compromise patient privacy.

Future research should look into subtype consensus for individual primary diagnoses in more details for carefully curated data sets. As well, the need for much larger curated archives in the pathology community is clearly evident, which includes additional tissue types such as hematological. Lastly, comprehensive discordance measurement for subtypes with and without computational consensus should be planned and carried out as the ultimate evidence for the efficacy of the image search as a supportive diagnostic tool.

The intellectual property as well as the financial implications for related works emerging from sharing image repositories are certainly significant issues that need elaboration in future works.

## Methods

### Data collection

We used the publicly available data set of 30,072 WSIs from the TCGA project^49,50^ (Genomic Data Commons GDC). Due to the retrospective nature of this study using only publicly available data, ethics approval was not required. All WSIs are tagged with a primary diagnosis. We removed 952 WSIs due to the following reasons: poor staining, low resolution, lack of all magnification levels in the WSI pyramid, large presence of out-of-focus regions, and/or presence of unreadable regions within an image. Most WSIs had a magnification of 20$\times$ or 40$\times$, some at lower magnifications. In total, we processed 29,120 WSIs at 20$\times$ magnification (approximately six terabytes in compressed form) for this study. The data set contains 25 anatomic sites with 32 cancer subtypes. Ten tumor types (brain, endocrine, gastrointestinal tract, gynecological, hematopoietic, liver/pancreaticobiliary, melanocytic, prostate/testis, pulmonary, and urinary tract) had more than one primary diagnoses. From the 29,120 WSIs, 26,564 specimens were neoplasms, and 2556 were non-neoplastic. A total of 17,425 files comprised frozen section digital slides, and 11,579 files were of permanent hematoxylin and eosin (H&E) sections. For the remaining 116 WSIs, the tissue section preparation was unspecified. We did not remove manual pen markings from the slides when present. The TCGA codes for all 32 cancer subtypes are provided in Table 5 in the appendix. The TCGA data set has a number of shortcomings^50^. Many of the cases are of frozen section in which tissue morphology may be compromised by frozen artifacts. Available cases may also reflect research bias in institutional biorepository collections. Furthermore, “tumors routinely subjected to neoadjuvant therapy may not have been able to be included in TCGA, because of limited availability of untreated specimens”^50^. Moreover, hematopathology is conspicuously absent from the TCGA data"set with just a few lymph nodes included. In spite of the shortcomings, the TCGA is the largest public data set that can support a pan-cancer validation of AI solutions for digital pathology.

**Table 5:** The TCGA codes (in alphabetical order) of all 33 primary diagnoses and corresponding number of evidently diagnosed patients in the data set (TCGA = The Cancer Genome Atlas).

TCGA Code	Primary diagnosis	Number of patients
ACC	Adrenocortical carcinoma	86
BLCA	Bladder urothelial carcinoma	410
BRCA	Breast invasive carcinoma	1097
CESC	Cervical squamous cell carcinoma and endocervical adenocarcinoma	304
CHOL	Cholangiocarcinoma	51
COAD	Colon adenocarcinoma	459
DLBC	Lymphoid neoplasm diffuse large B-cell lymphoma	48
ESCA	Esophageal carcinoma	185
GBM	Glioblastoma multiforme	604
HNSC	Head and neck squamous cell carcinoma	473
KICH	Kidney chromophobe	112
KIRC	Kidney renal clear cell carcinoma	537
KIRP	Kidney renal papillary cell carcinoma	290
LGG	Brain lower-grade glioma	513
LIHC	Liver hepatocellular carcinoma	376
LUAD	Lung adenocarcinoma	522
LUSC	Lung squamous cell carcinoma	504
MESO	Mesothelioma	86
OV	Ovarian serous cystadenocarcinoma	590
PAAD	Pancreatic adenocarcinoma	185
PCPG	Pheochromocytoma and paraganglioma	179
PRAD	Prostate adenocarcinoma	499
READ	Rectum adenocarcinoma	170
SARC	Sarcoma	261
SKCM	Skin cutaneous melanoma	469
STAD	Stomach adenocarcinoma	442
TGCT	Testicular germ cell tumors	150
THCA	Thyroid carcinoma	507
THYM	Thymoma	124
UCEC	Uterine corpus endometrial carcinoma	558
UCS	Uterine carcinosarcoma	57
UVM	Uveal melanoma	80

### The search algorithm

**Fig. 9:**
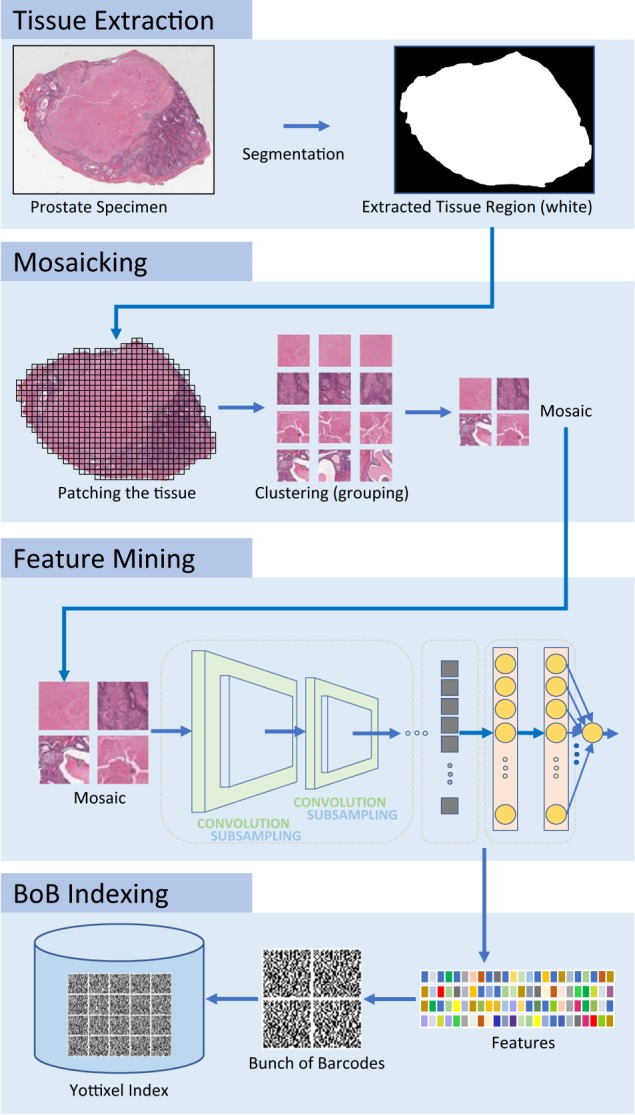
Yottixel image search engine: whole-slide images are segmented first to extract the tissue region by excluding the background (top block). A mosaic of representative patches (tiles) is assembled through grouping of all patches of the tissue region using an unsupervised clustering algorithm (second block from the top). All patches of the mosaic are fed into a pretrained artificial neural network for feature mining (third block from the top). Finally, a bunch of barcodes is generated and added to the index of all WSI files in the archive (bottom block).

The Yottixel image search engine incorporates clustering, transfer learning, and barcodes and was used to conduct all experiments^30,32,41–43,51–54^. Before any search can be performed, all images in the repository have to be “indexed”, i.e., every WSI is catalogued utilizing a “bunch of barcodes” (BoB indexing). These barcodes are stored for later use and generally not visible to the user. This process contains several steps (Fig. 9):

- Tissue extraction—Every WSI contains a bright (white) background that generally contains irrelevant (non-tissue) pixel information. In order to process the tissue, we need to segment the tissue region(s), and generate a black and white image (binary mask) that provides the location of all tissue pixels as “1” (white). Such a binary mask is depicted in the top row of Fig. 9.
- Mosaicking—Segmented tissue now gets patched (divided into patches/tiles). These patches have a fixed size at a fixed magnification (e.g., 500 × 500 µm^2^ at 20$\times$ scan resolution). All patches of the WSI get grouped into a pre-set number of categories (classes) via a clustering method (we used $k$-means algorithm^55^). A clustering algorithm is an unsupervised method that automatically groups WSI patches into clusters (i.e., groups) that contain similar tissue patterns. A small percentage (5–20%) of all clustered patches are selected uniformly distributed within each class to assemble a mosaic. This mosaic represents the entire tissue region within the WSI. A sample mosaic consisting of four patches is depicted in the second row of Fig. 9. Most WSIs we processed had a mosaic with around 70–100 patches.
- Feature mining—All patches of the mosaic of each WSI are now pushed through pretrained artificial neural networks (generally trained with natural images using data sets such as ImageNet^56^). The output of the network is ignored and the last pooling layers or the first connected layers are generally used as “features” to represent each mosaic patch. There could be ~1000–4000 features. The third row of Fig. 9 shows this process where the features (colored squares) are passed on to the next stage, namely BoB indexing.
- Bunch of barcodes—All feature vectors of each mosaic are subsequently converted into binary vectors using the *MinMax* algorithm^43^. This bunch of barcodes is the final index information for every query/input WSI that will be stored in the Yottixel index for future or immediate search. This is illustrated at the bottom of Fig. 9.

In summary, Yottixel assigns “a bunch of barcodes” to each WSI to index the entire digital slide. The BoB indexing enables Yottixel to search a large archive of histopathology images very efficiently. The index can be easily shared among institutions if necessary. Technical details of Yottixel algorithms are described in a separate paper where its performance was tested with 2300 WSIs^41^.

### Reproducibility

Does image search generate the same results for the same WSI if fed into the Yottixel engine again? We ran indexing several times and the results did not change significantly. We observed slight changes in the order of search results affecting neither the hit rate nor the majority vote. The only component of our approach with some non-deterministic behavior is the K-means clustering algorithm. However, the K-means is run for as many iterations until it converges to a stable solution when we index WSIs. After a new WSI has been indexed its “bunch of barcodes” do not change anymore, and hence the same WSI as input (with unique patient ID) will generate the same results.

### Reporting summary

Further information on research design is available in the Nature Research Reporting Summary linked to this article.

## Supplementary information

Reporting Summary

## Data Availability

The publicly available data set of 30,072 WSIs from the TCGA project^49,50^ (Genomic Data Commons GDC) is used for conducting this study.

## Code availability

The deep models used in this study were implemented using TensorFlow. The weights for pretrained models are openly provided by Keras libray. Statistical analysis was performed using Python libraries—Scikit Learn, Numpy, and Pandas. The Dask library was used as distributed computing framework for running large-scale computation jobs.
